# Supplementary material for: Empirical methods for controlling false positives and estimating confidence in ChIP-Seq peaks
Source: BMC Bioinformatics. 2008 Dec 5;9:523. doi: 10.1186/1471-2105-9-523 (PMC2628906; doi:10.1186/1471-2105-9-523)
Supplement: Additional File 1 — A variety of html documents from the USeq web site detailing the available applications, their best usage, output file type descriptions, command line menus, etc. [file 1471-2105-9-523-S1.zip › USeqUserGuides/JavaDoc.zip!/JavaDoc/trans/main/WilcoxonSamplePair.html]

WilcoxonSamplePair


|  |  |  |
| --- | --- | --- |
| **Overview** \| **Package** \| **Class** \| **Use** \| **Tree** \| **Deprecated** \| **Index** \| **Help** | |  |
| **PREV CLASS**   **NEXT CLASS** | **FRAMES**    **NO FRAMES**     **All Classes** |  |
| SUMMARY: NESTED \| FIELD \| CONSTR \| METHOD | DETAIL: FIELD \| CONSTR \| METHOD |  |


---


## trans.main Class WilcoxonSamplePair

```
java.lang.Object
  trans.main.WilcoxonSamplePair
```

**All Implemented Interfaces:**: java.lang.Comparable

---

``` public class WilcoxonSamplePair extends java.lang.Object implements java.lang.Comparable ```

Used by the `WilcoxonSignedRankTest`.

---

**Constructor Summary**

`WilcoxonSamplePair(float absoluteDifference)`

`WilcoxonSamplePair(float tValue,
float cValue)`


| **Method Summary** | |
| --- | --- |
| `int` | `compareTo(java.lang.Object obj)` |
| `float` | `getAbsoluteDifference()` |
| `float` | `getCValue()` |
| `float` | `getDifference()` |
| `float` | `getRank()` |
| `float` | `getTValue()` |
| `void` | `setAbsoluteDifference(int absoluteDifference)` |
| `void` | `setRank(float rank)` |
| `java.lang.String` | `toString()` |

**Methods inherited from class java.lang.Object**

`equals, getClass, hashCode, notify, notifyAll, wait, wait, wait`

**Constructor Detail**

### WilcoxonSamplePair

```
public WilcoxonSamplePair(float tValue,
                          float cValue)
```

---


### WilcoxonSamplePair

```
public WilcoxonSamplePair(float absoluteDifference)
```


**Method Detail**

### compareTo

```
public int compareTo(java.lang.Object obj)
```

:   **Specified by:**: `compareTo` in interface `java.lang.Comparable`

---


### toString

```
public java.lang.String toString()
```

:   **Overrides:**: `toString` in class `java.lang.Object`

---


### getAbsoluteDifference

```
public float getAbsoluteDifference()
```

---


### getCValue

```
public float getCValue()
```

---


### getDifference

```
public float getDifference()
```

---


### getRank

```
public float getRank()
```

---


### getTValue

```
public float getTValue()
```

---


### setAbsoluteDifference

```
public void setAbsoluteDifference(int absoluteDifference)
```

---


### setRank

```
public void setRank(float rank)
```


---


|  |  |  |
| --- | --- | --- |
| **Overview** \| **Package** \| **Class** \| **Use** \| **Tree** \| **Deprecated** \| **Index** \| **Help** | |  |
| **PREV CLASS**   **NEXT CLASS** | **FRAMES**    **NO FRAMES**     **All Classes** |  |
| SUMMARY: NESTED \| FIELD \| CONSTR \| METHOD | DETAIL: FIELD \| CONSTR \| METHOD |  |


---
